# Supplementary material for: GPR180 deficiency impairs mitochondrial function and insulin secretion in pancreatic β-cells
Source: Mol Metab. 2026 Jul 16;111:102420. doi: 10.1016/j.molmet.2026.102420 (PMC13417978; doi:10.1016/j.molmet.2026.102420)
Supplement: Multimedia component 1 [file mmc1.docx]

**Methods details**

*Gene expression analysis in mouse pancreatic islets and MIN6 cells*

For transcriptomic analysis, total RNA was extracted from isolated pancreatic islets of Langerhans using a phenol-chloroform method with DNase treatment to eliminate genomic DNA contamination. RNA quantity was measured by Qubit fluorometry (Invitrogen), and integrity was confirmed with an Agilent 2100 Bioanalyzer. Libraries were prepared from >100 ng input RNA using poly(A) enrichment and directional library construction. mRNA sequencing was performed by Novogene Co., Ltd. (Munich, Germany) on an Illumina NovaSeq 6000 platform with paired-end 150 bp reads. Data output was ≥ 20 million read pairs per sample, ensuring comprehensive transcriptome coverage. Raw sequencing data were processed using Novogene’s in-house bioinformatics pipeline, NovoMagic, which includes quality filtering, alignment to the reference genome, transcript assembly, gene expression quantification, and differential expression analysis. Differentially expressed genes (DEGs) were defined by |log2(Fold Change)| ≥ 1 and adjusted p-value (padj) ≤ 0.05. Functional enrichment analyses (GO and KEGG) applied a significance threshold of padj < 0.05. We acknowledge Novogene for providing high-quality sequencing and bioinformatics services.

Knockout efficiency in isolated islets was confirmed by qPCR. First, extracted total RNA was reversed transcribed using the High Capacity cDNA Reverse transcription kit (Applied Biosystems), with 200 ng of RNA. Gene expression of *Gpr180* was normalized to housekeeping gene *Tbp*.

For mitochondrial DNA copy number, genomic DNA was extracted from the organic phase following RNA isolation from pancreatic islets using Back Extraction Buffer (4M GTC, 50 mM Na₃C₆H₅O₇, 1M Tris). DNA concentration and purity were measured spectrophotometrically with a Nanodrop (ThermoFisher Scientific), and samples were diluted to a uniform concentration prior to analysis. Relative mitochondrial DNA content was determined by normalizing expression of the mitochondrial gene *Nd1* to the nuclear reference gene *Lpl.*

Quantitative real-time PCR was performed in technical duplicates using Fast SYBR Green Master Mix (Applied Biosystems) on a QuantStudio 5 system (ThermoFisher Scientific). All primer sequences are provided in Supplementary Table 1.

Gene expression analysis in MIN6 cells (standard culture conditions), particularly *t*otal RNA extraction, qPCR to determine knockdown as well as overexpression efficiency, mitochondrial copy number determination and RNAseq was performed as described above for isolated islets.

*Metabolomics*

MIN6 cells cultured in standard medium 48h post-transfection were quenched and deproteinized on dry ice by adding 200 µL of ice-cold 80% methanol containing internal standards (10 µM creatinine-D3, 10 µM leucine-D3, 0.5 µM methylmalonate-D3, 0.1 µM butyrylcarnitine-D3, and 45 µM lactate-D3), followed by vortex mixing and sonication. After overnight protein precipitation at −80 °C, the samples were briefly vortexed and centrifuged. The supernatant from each sample was split into two aliquots: one part was used for wide-coverage targeted metabolomic analysis, and the other portion was evaporated to dryness and reconstituted in water for organic acid (OA) analysis. Equal aliquots from all samples were pooled to prepare the quality control (QC) sample. Metabolomic and OA analyses were performed using a Nexera LC system (Sciex, Framingham, MA, USA) coupled to a QTRAP 6500+ mass spectrometer (Sciex, Framingham, MA, USA). Both methods were carried out in positive and negative ion modes with polarity switching using scheduled multiple reaction monitoring (sMRM). Detailed information of the targeted metabolomic [1], and organic acid [2] methods, including chromatographic conditions and mass spectrometry settings, are provided in our previously published work.

Raw data from the metabolomic and OA analyses were acquired using SCIEX OS (v4.0) and Analyst software (v1.7.3) (SCIEX, Framingham, MA, USA). Data processing and statistical evaluation were performed in R (v4.5.1, [www.r-project.org](http://www.r-project.org/)) using the Metabol package [3]. Local estimated smoothing signal (LOESS) correction was applied to each dataset independently. Metabolites with a coefficient of variation greater than 30% in QC samples were excluded from further analysis. The datasets were merged prior to statistical evaluation, and a natural logarithmic transformation was applied. Results were visualized using box plots, a Cytoscape metabolic map (Cytoscape v3.10.4, Bethesda, MD, USA), and MetaboAnalyst software (v6.0) [4]. Within Cytoscape, node size corresponded to the −log(p-value) derived from the Student’s t-test, whereas node color indicated the fold change, with red denoting an increase and blue a decrease in GPR180-deficient cells relative to controls. In MetaboAnalyst, Pareto scaling was applied to the data structure. In the pathway analysis, the x-axis represents the pathway impact score, whereas the y-axis shows the −log(p-value) obtained from the enrichment analysis. The enrichment analysis generated a ranked set of pathways affected in the dataset, visualizing the top 25 pathways according to their statistical relevance.

*Mitochondrial respiration*

MIN6 cells were plated, and transfections were performed on 96-well Seahorse microplates. On the day of the experiment, the standard culture medium was replaced with XF DMEM Medium (pH 7.4, Agilent) supplemented with glucose (25 mM; Sigma–Aldrich), 2 mM sodium pyruvate (Invitrogen), and 2 mM GlutaMax (Invitrogen). Test compounds were sequentially injected to achieve the following final concentrations: 1.25 µM Oligomycin, 10 µM FCCP, 3 µM Rotenone with 3.75 µM Antimycin A. All compounds were purchased from Sigma–Aldrich. The oxygen consumption rate (OCR) was measured using the Seahorse XF Pro Analyzer and analyzed by Wave Pro software (Agilent Seahorse). OCR levels (pmol/min) were normalized to protein content per well (µg protein). Non-mitochondrial respiration was subtracted to obtain basal, coupled, uncoupled (proton leak) and maximal mitochondrial respiration.

*Measurement of intracellular ATP levels*

Cells were washed 48 hours post-transfection and preincubated for 1 hour in KRB (129 mM NaCl, 4.8 mM KCl, 1 mM CaCl_2_, 5 mM NaHCO_3_, 1.2 mM KH_2_PO4, 1.2 mM MgSO_4_, 10 mM HEPES, 0.1% BSA, pH 7.4) containing low glucose (2 mM). Subsequently, cells were incubated for 1 hour under either basal (2 mM glucose) or stimulatory (25 mM glucose) conditions. To inhibit mitochondrial pyruvate uptake, cells were treated with 2 µM UK-5099, a specific inhibitor of the mitochondrial pyruvate carrier. To inhibit the entry of fatty acids bound to albumin present in the assay buffer into mitochondria, 100 µM etomoxir was used. Glucose metabolism was blocked by the addition of 2-deoxyglucose. All compounds were purchased from Sigma–Aldrich. Finally, culture supernatant was quickly discarded and cells were lysed in Passive lysis buffer (Promega). Intracellular ATP concentrations were quantified in cell lysates using a bioluminescence-based ATP Determination Kit (Invitrogen), following the manufacturer’s protocol. ATP levels were normalized to protein content determined by Pierce™ BCA Protein Assay Kits (ThermoFisher Scientific). Luminescence and absorbance were determined by SynergyMx Plate microplate reader (Biotek).

*Samples preparation for transmission electron microscopy*

MIN6 cells 48h post-transfection were mildly trypsinized with 0.05% Trypsin-EDTA for 2 min at room temperature (RT) and subsequently detached by tapping the plate in order to minimize changes in cell morphology. Cell suspension was centrifuged at 1000 rpm/5 min/RT and the cell pellet was fixed for 2 hours in 2% glutaraldehyde in cacodylate buffer and post-fixed in 1% osmium tetroxide (OsO₄) in the same buffer. After washing, samples were contrasted with 1% aqueous uranyl acetate. Dehydration was carried out through a graded ethanol series followed by propylene oxide, after which samples were infiltrated with a 1:1 mixture of propylene oxide and Durcupan resin. Polymerization was performed in pure Durcupan at 60 °C for 72 hours.

**References**

1. Cífková E, Brumarová R, Ovčačíková M, et al (2022) Lipidomic and metabolomic analysis reveals changes in biochemical pathways for non-small cell lung cancer tissues. Biochim Biophys Acta Mol Cell Biol Lipids 1867(2):159082. https://doi.org/10.1016/j.bbalip.2021.159082

2. Piskláková B, Friedecká J, Ivanovová E, et al (2023) Rapid and efficient LC-MS/MS diagnosis of inherited metabolic disorders: a semi-automated workflow for analysis of organic acids, acylglycines, and acylcarnitines in urine. Clin Chem Lab Med 61(11):2017–2027. https://doi.org/10.1515/cclm-2023-0084

3. AlzbetaG (2019) AlzbetaG/Metabol: First version; DOI:10.5281/zenodo.3235775

4. Pang Z, Lu Y, Zhou G, et al (2024) MetaboAnalyst 6.0: towards a unified platform for metabolomics data processing, analysis and interpretation. Nucleic Acids Res 52(W1):W398–W406. https://doi.org/10.1093/nar/gkae253
