# Supplementary material for: GPR180 deficiency impairs mitochondrial function and insulin secretion in pancreatic β-cells
Source: Mol Metab. 2026 Jul 16;111:102420. doi: 10.1016/j.molmet.2026.102420 (PMC13417978; doi:10.1016/j.molmet.2026.102420)
Supplement: Multimedia component 2 [file mmc2.docx]

| **gene** | **Fwd primer sequence** | **Rev primer sequence** |
| --- | --- | --- |
| *Gpr180* | CGCAGTCTTCATCGTCATCA | CTGTGGTGACTGGTCTCTTCA |
| *GPR180* | GCATCGGCCACTTCGAGTTC | TGGGCTTGGAACAGGTAGAGT |
| *gLpl* | GGATGGACGGTAAGAGTGATTC | ATCCAAGGGTAGCAGACAGGT |
| *mt-Nd1* | CAGCCTGACCCATAGCCATAATAT | TGATTCTCCTTCTGTCAGGTCGAA |
| *Tbp* | GAAGCTGCGGTACAATTCCAG | CCCCTTGTACCCTTCACCAAT |

**Supplementary Table 1: List of primers used in qPCR.**
