# Supplementary material for: GPR180 deficiency impairs mitochondrial function and insulin secretion in pancreatic β-cells
Source: Mol Metab. 2026 Jul 16;111:102420. doi: 10.1016/j.molmet.2026.102420 (PMC13417978; doi:10.1016/j.molmet.2026.102420)
Supplement: Multimedia component 3 [file mmc3.docx]

| **target** | **dilution** | **vendor** | **RRID** |
| --- | --- | --- | --- |
| AMPK, phosphorylated at Thr172 | 1 : 1000 | Cell Signaling | AB_331250 |
| AMPK, total | 1 : 1000 | Cell Signaling | AB_10622186 |
| gamma tubulin | 1 : 10 000 | Sigma–Aldrich | AB_477584 |
| HSP90 | 1 : 1000 | Cell Signaling | AB_2233307 |
| p44/42 MAPK (Erk1/2), phosphorylated at Thr202/Tyr204 | 1 : 1000 | Cell Signaling | AB_2315112 |
| OXPHOS | 1 : 500 | Abcam | AB_2629281 |
| Phospho-PKA Substrate (RRXS*/T*) | 1 : 1000 | Cell Signaling | AB_331817 |
| [Phospho-(Ser) PKC Substrate Antibody](https://rrid.site/data/record/nif-0000-07730-1/RRID:AB_330310/resolver?q=pkc%20substrates&i=rrid:ab_330310-98661) | 1 : 1000 | Cell Signaling | AB_330310 |
| [Smad3, phosphorylated at Ser423 and S425](https://rrid.site/data/record/nif-0000-07730-1/RRID:AB_882596/resolver?q=smad3&i=rrid:ab_882596-2179856) | 1 : 1000 | Abcam | AB_882596 |
| [IRDye 680RD Donkey anti-Mouse IgG](https://rrid.site/data/record/nif-0000-07730-1/RRID:AB_10953628/resolver?q=*&i=rrid:ab_10953628-1010534) | 1:10 000 | LI-COR | AB_10953628 |
| [IRDye 800CW Goat anti-Rabbit IgG](https://rrid.site/data/record/nif-0000-07730-1/RRID:AB_621843/resolver?q=*&i=rrid:ab_621843-180455) | 1:10 000 | LI-COR | AB_621843 |

**Supplementary Table 2: List of antibodies used in western blot.**
