# Supplementary figures and images for: GPR180 deficiency impairs mitochondrial function and insulin secretion in pancreatic β-cells
Source: Mol Metab. 2026 Jul 16;111:102420. doi: 10.1016/j.molmet.2026.102420 (PMC13417978; doi:10.1016/j.molmet.2026.102420)

Supplementary Figure 1

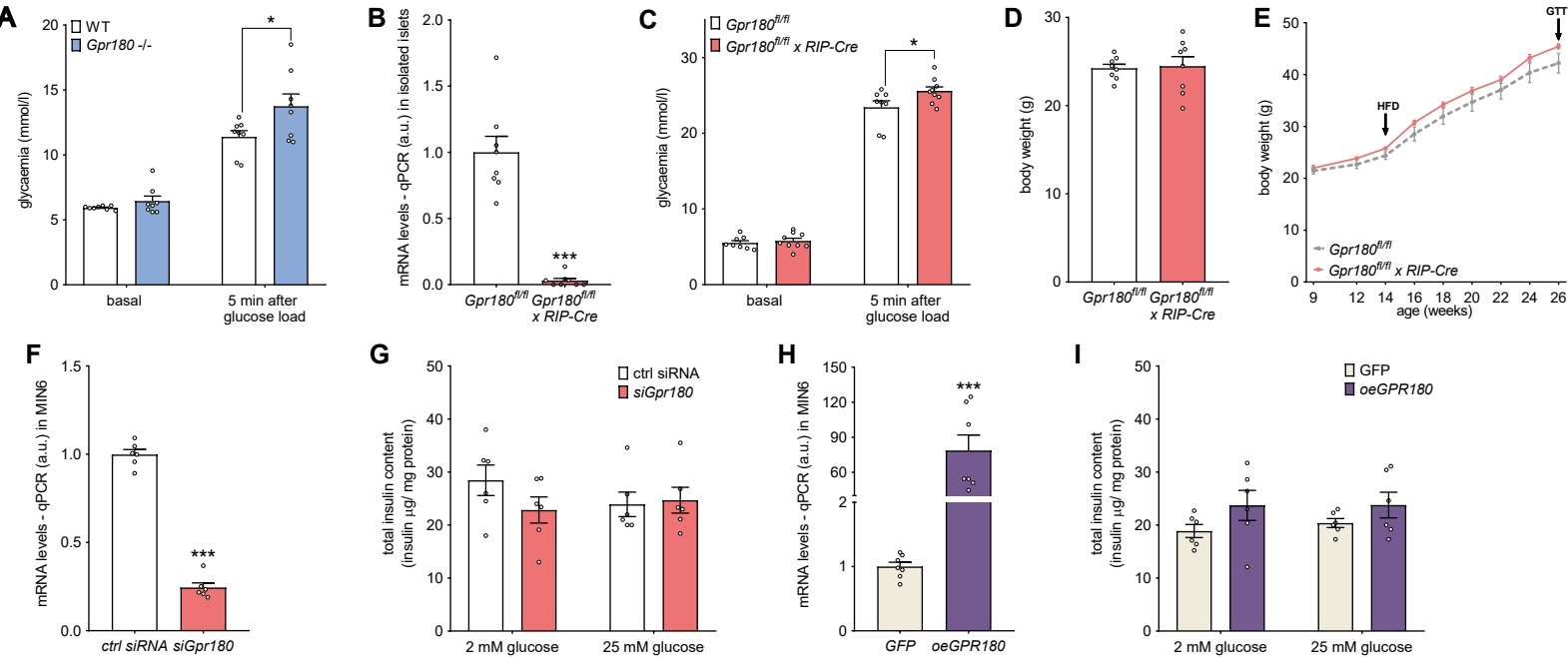

Supplement: Multimedia component 6 [file mmc6.pdf]

## Supplementary Figure 2

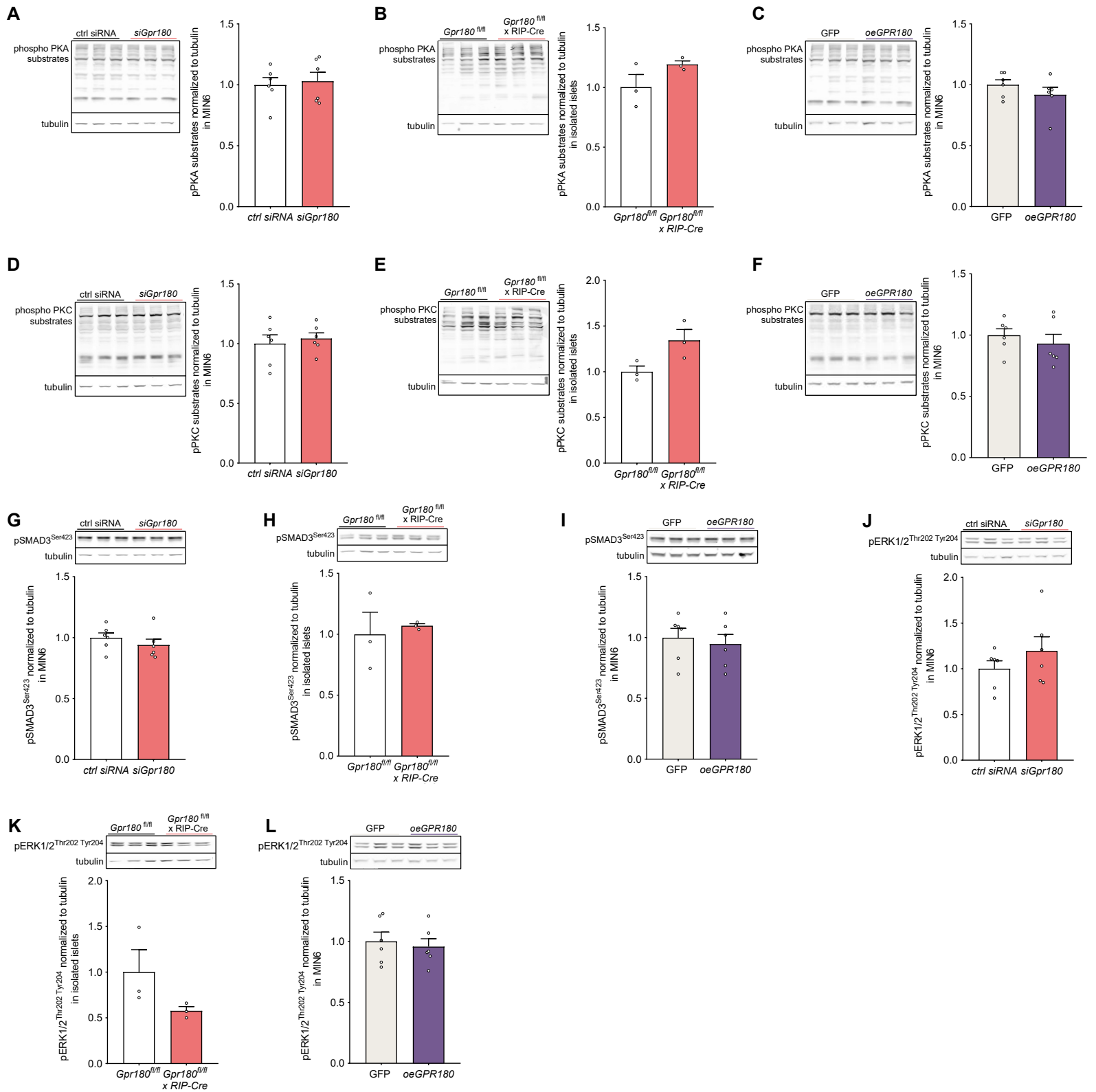

Supplement: Multimedia component 7 [file mmc7.pdf]

Supplementary Figure 3

A

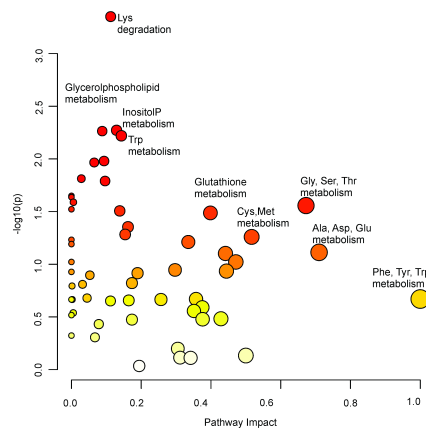

B

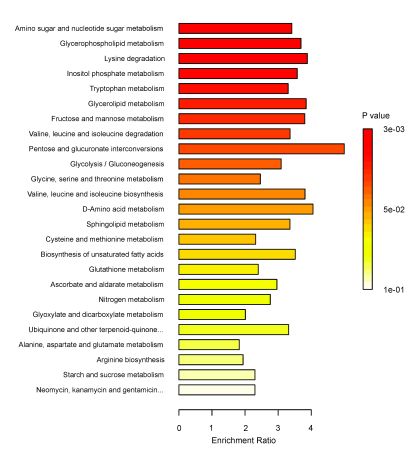

C

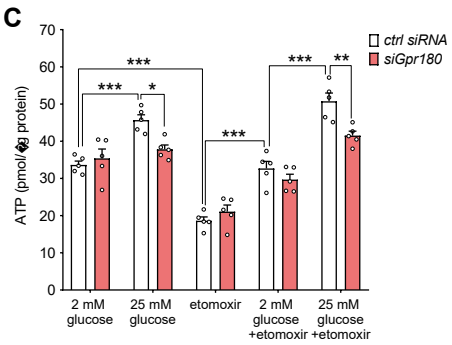

Supplement: Multimedia component 8 [file mmc8.pdf]

Supplementary Figure 4

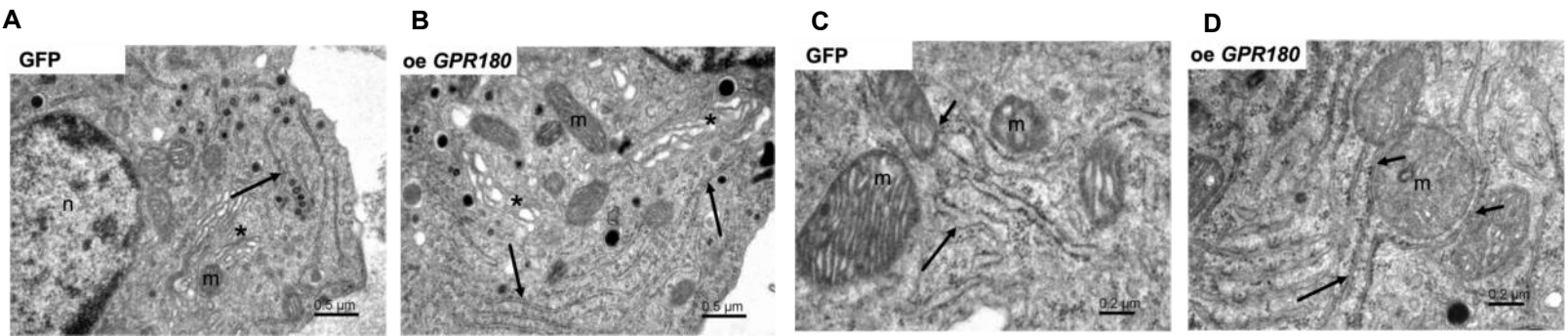

Supplement: Multimedia component 9 [file mmc9.pdf]
