# Supplementary material for: GPR180 deficiency impairs mitochondrial function and insulin secretion in pancreatic β-cells
Source: Mol Metab. 2026 Jul 16;111:102420. doi: 10.1016/j.molmet.2026.102420 (PMC13417978; doi:10.1016/j.molmet.2026.102420)
Supplement: Multimedia component 10 [file mmc10.pptx]

## Slide 1
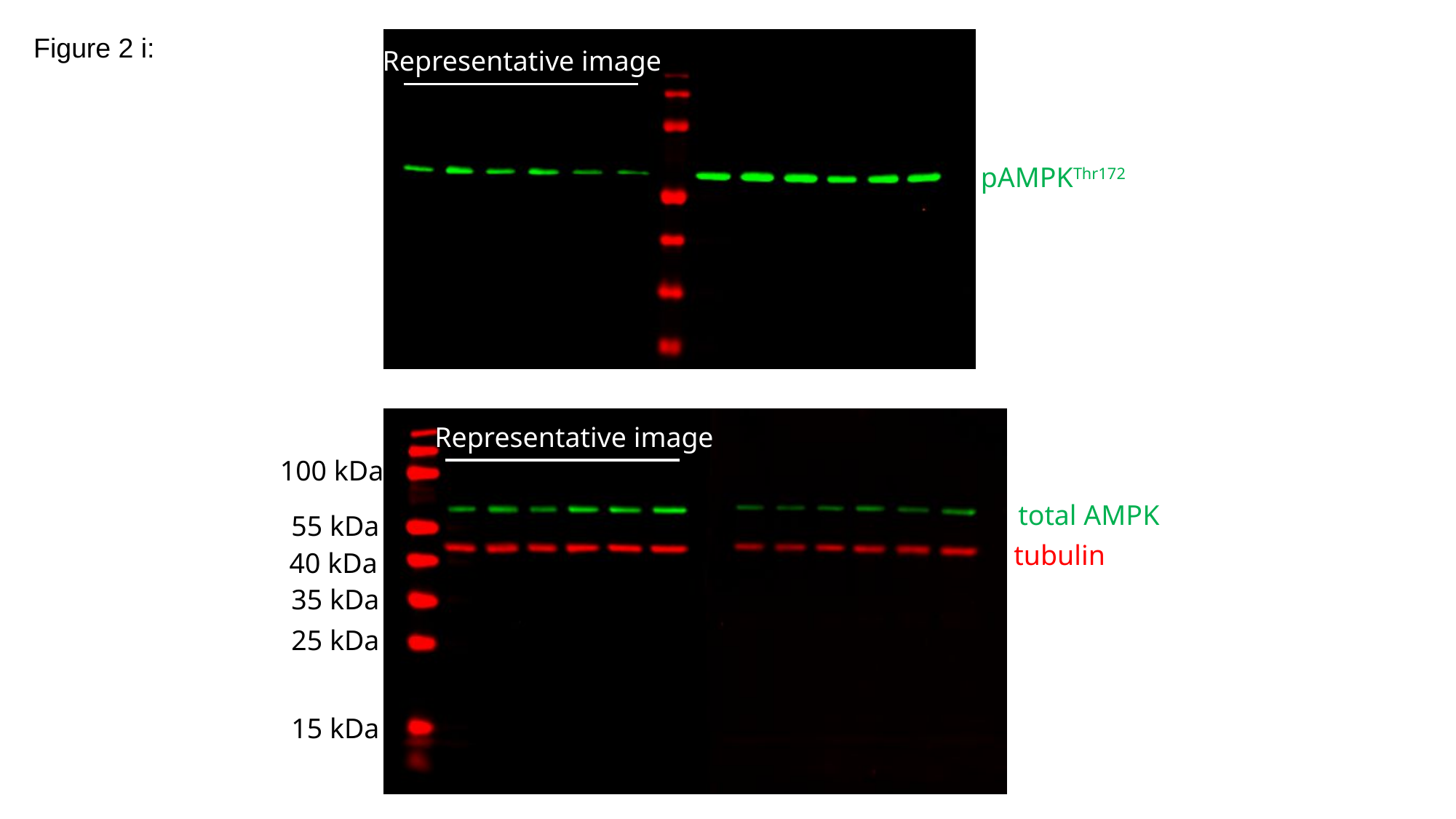

Figure 2 i:
pAMPKThr172
Representative image
Representative image
100 kDa
 total AMPK
55 kDa
tubulin
40 kDa
35 kDa
25 kDa
15 kDa

## Slide 2
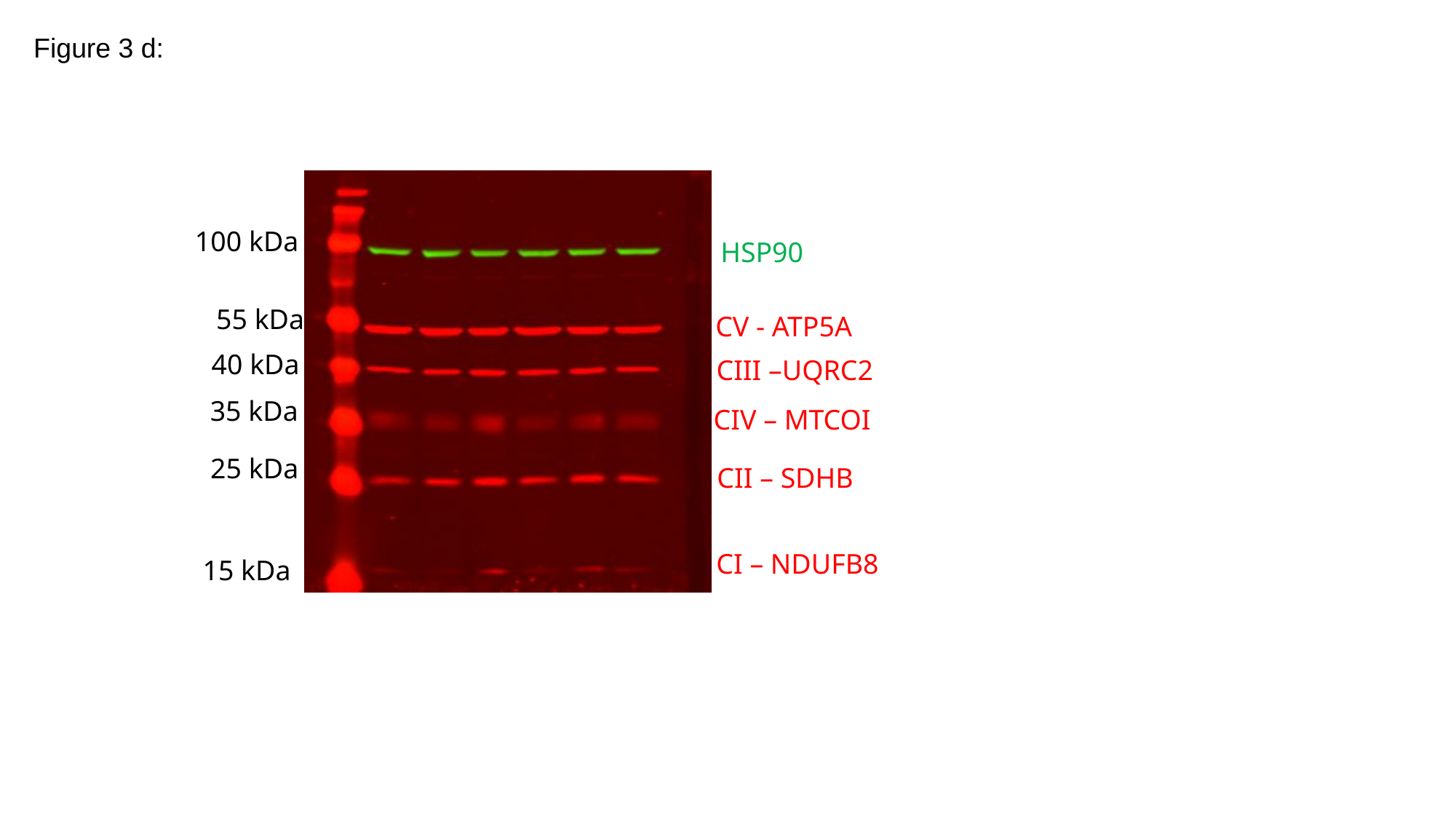

Figure 3 d:
100 kDa
55 kDa
CV - ATP5A
40 kDa
CIII –UQRC2
35 kDa
CIV – MTCOI
25 kDa
CII – SDHB
CI – NDUFB8
15 kDa
HSP90

## Slide 3
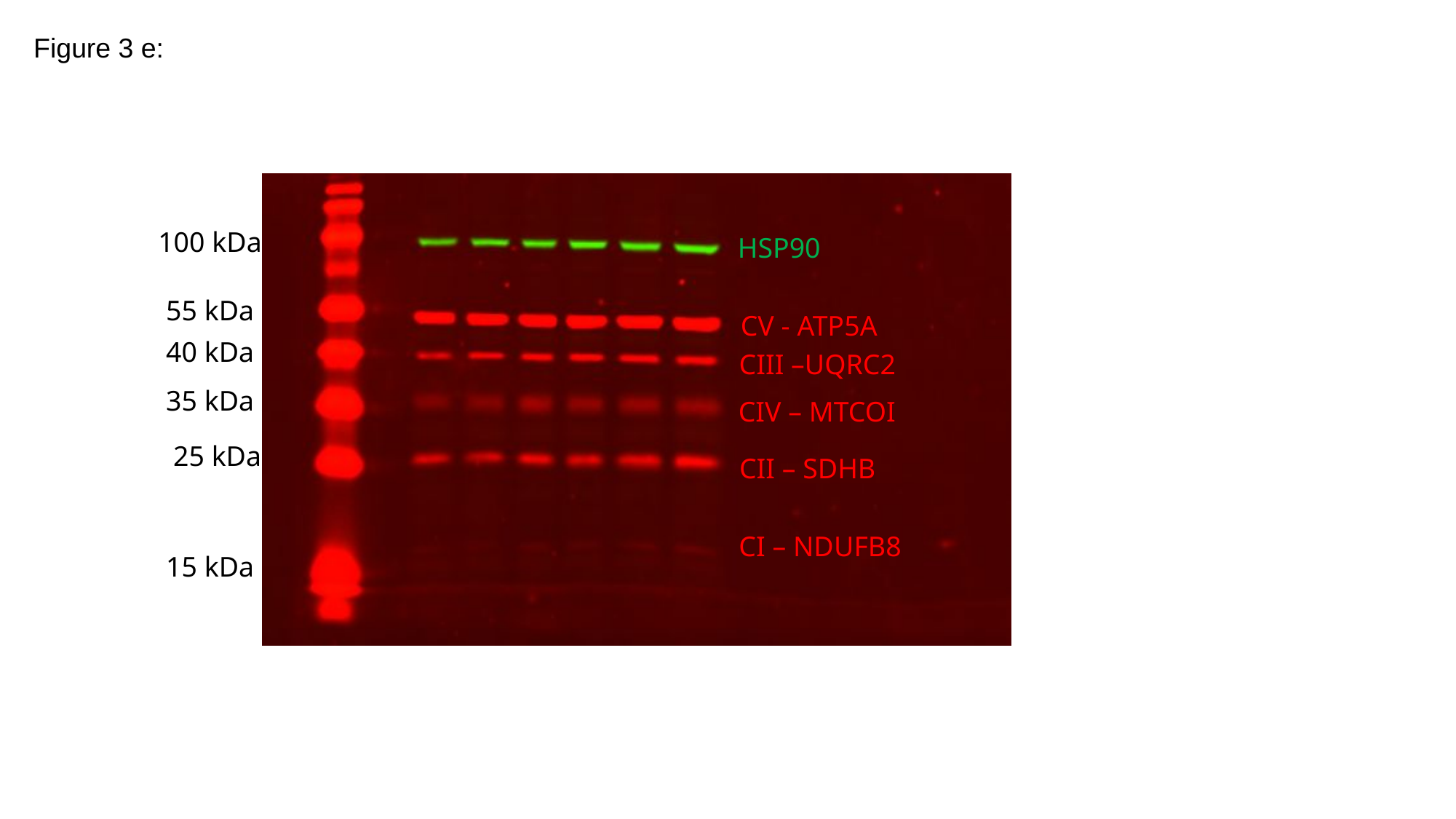

Figure 3 e:
100 kDa
55 kDa
CV - ATP5A
40 kDa
CIII –UQRC2
35 kDa
CIV – MTCOI
25 kDa
CII – SDHB
CI – NDUFB8
15 kDa
HSP90

## Slide 4
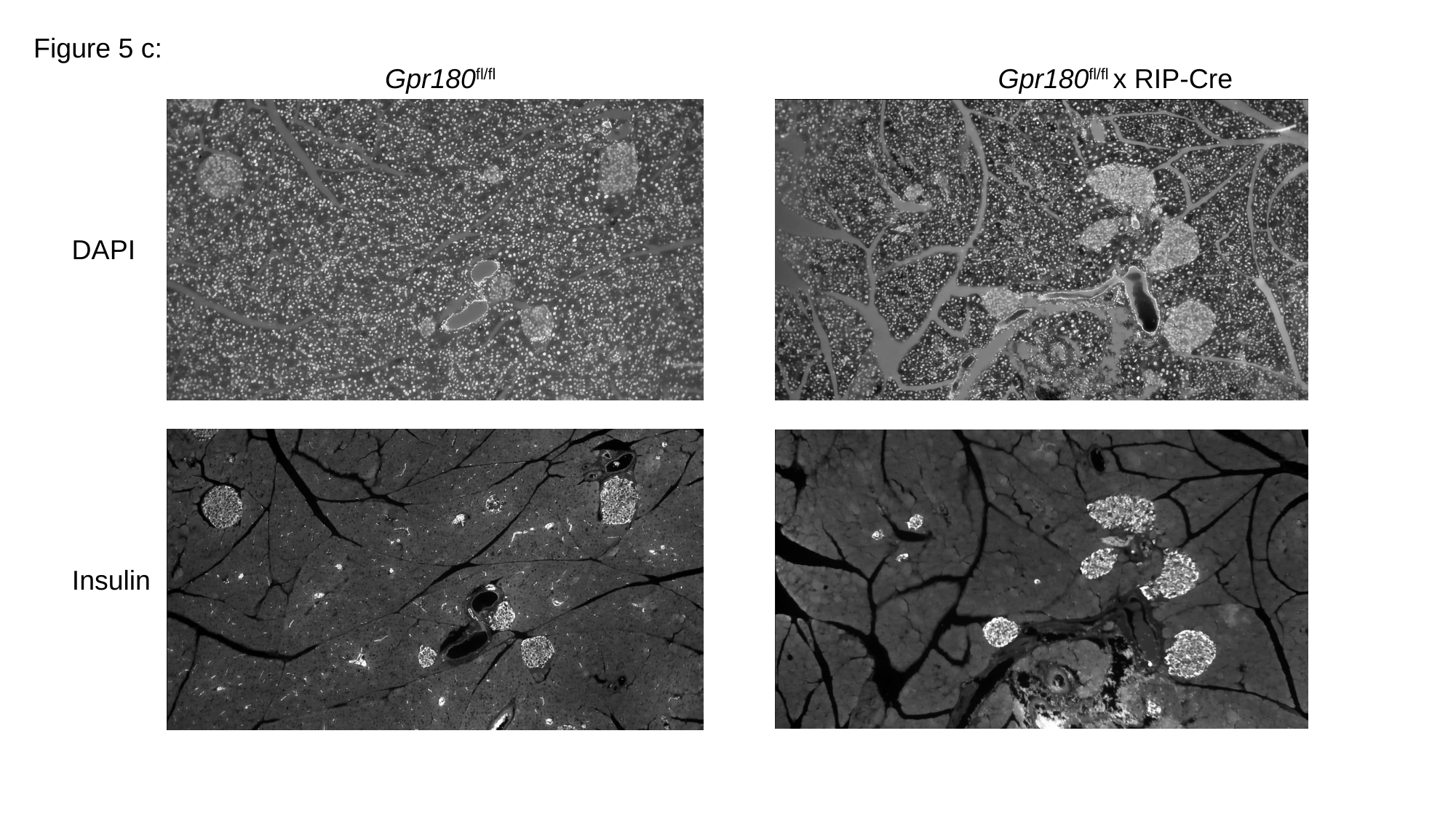

Figure 5 c:
Gpr180fl/fl
Gpr180fl/fl x RIP-Cre
DAPI
Insulin

## Slide 5
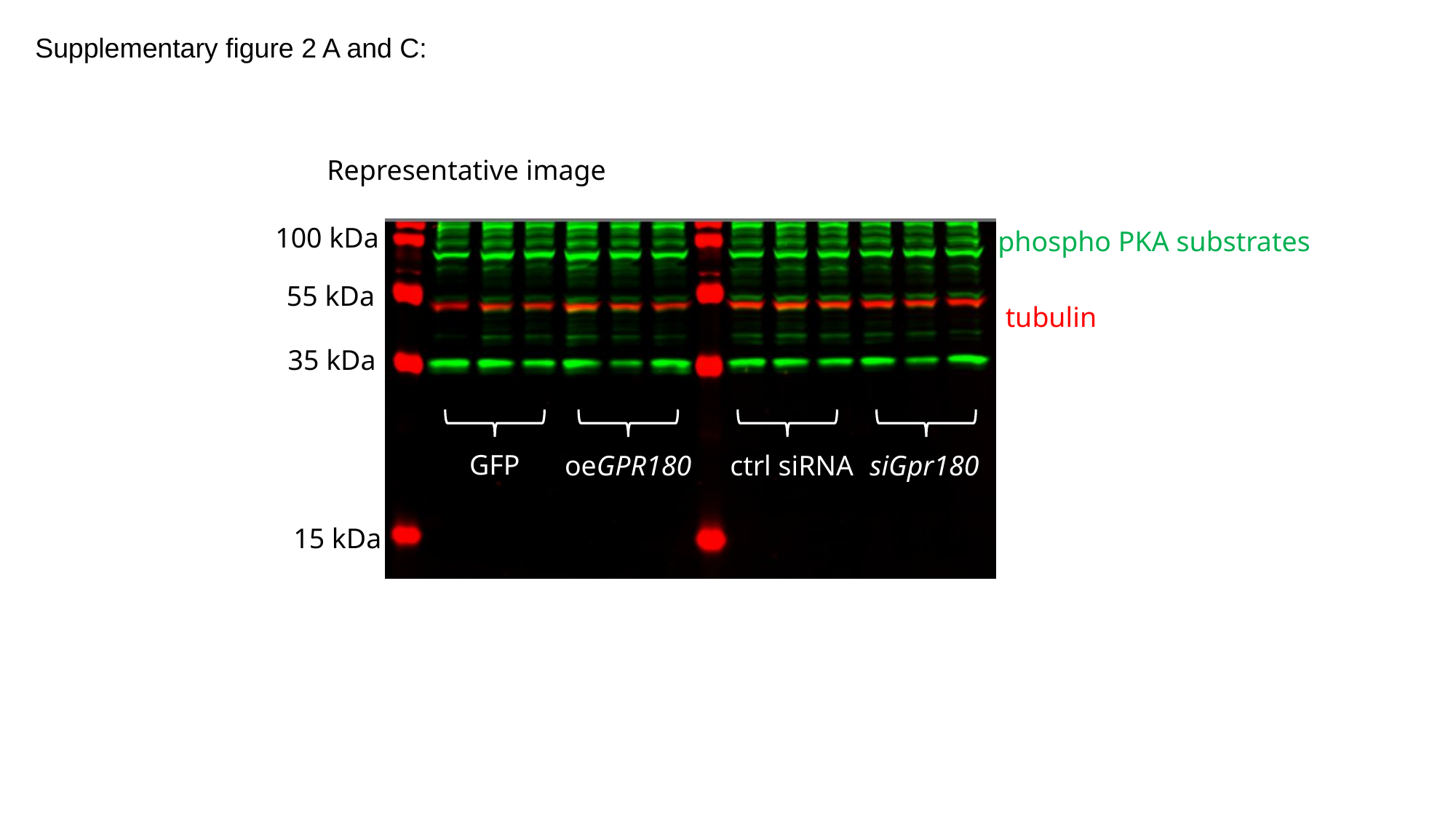

Supplementary figure 2 A and C:
phospho PKA substrates
Representative image
100 kDa
55 kDa
tubulin
35 kDa
15 kDa
GFP
oeGPR180
ctrl siRNA
siGpr180

## Slide 6
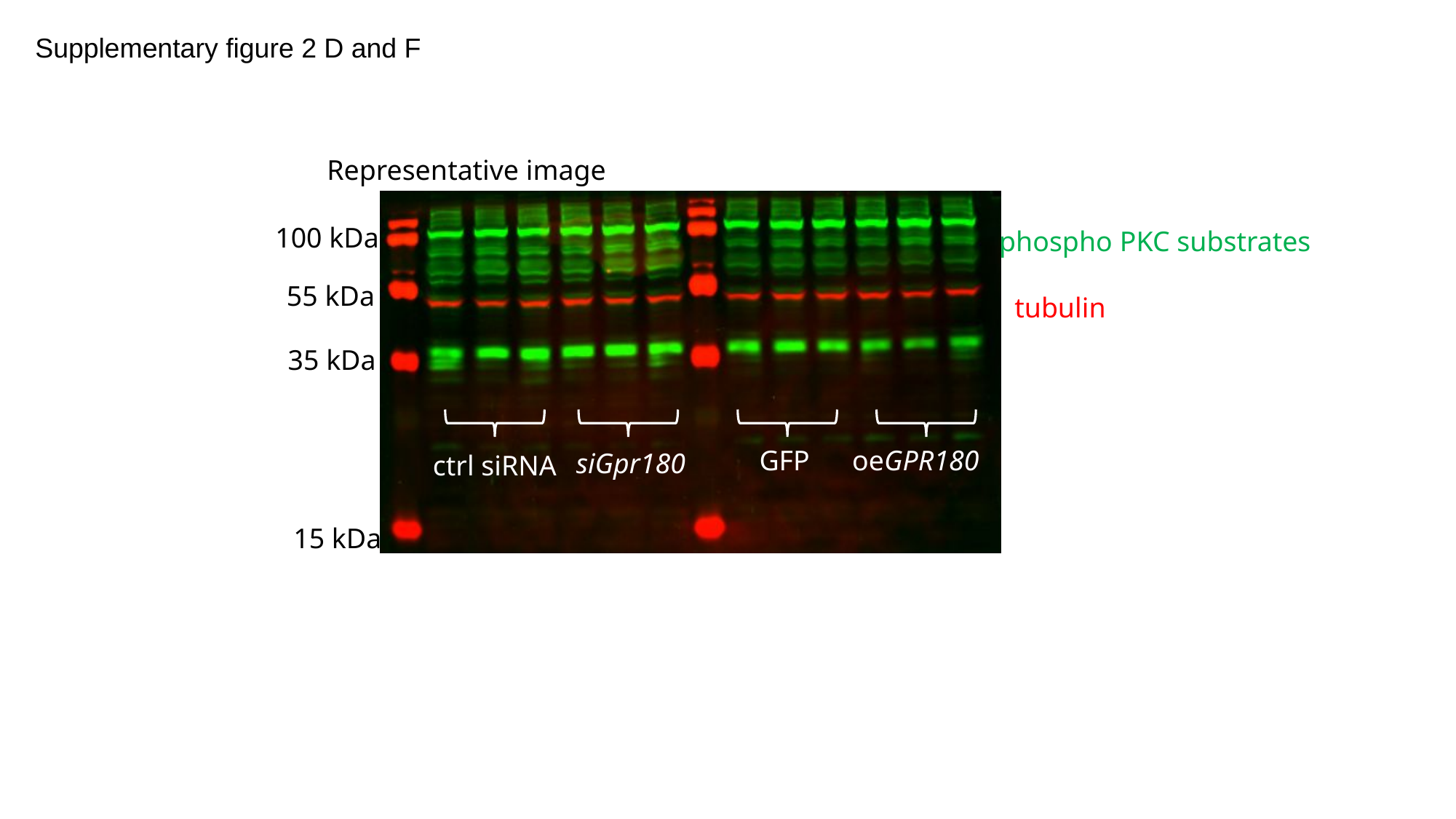

Supplementary figure 2 D and F
phospho PKC substrates
Representative image
100 kDa
55 kDa
tubulin
35 kDa
15 kDa
GFP
oeGPR180
siGpr180
ctrl siRNA

## Slide 7
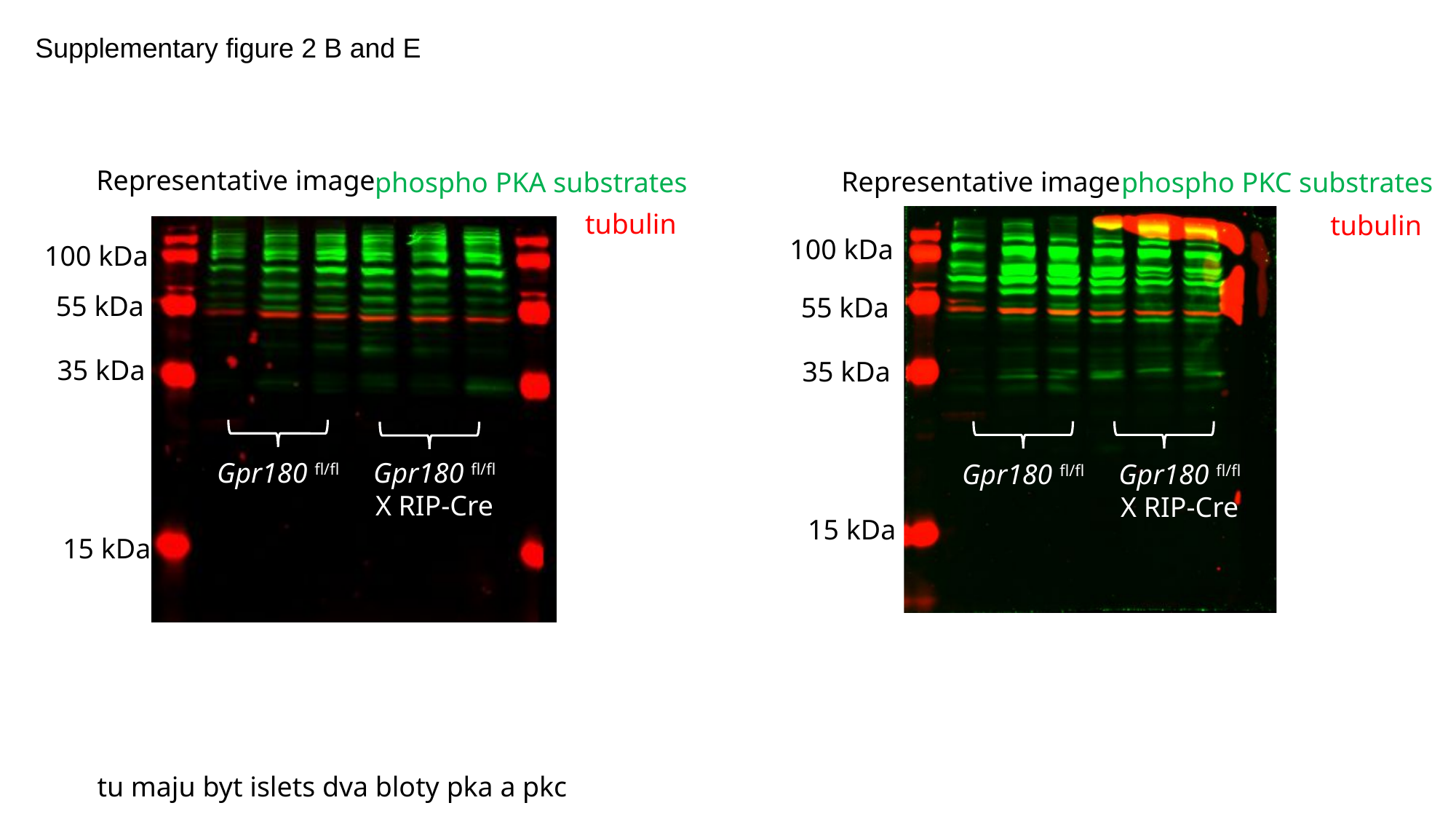

Supplementary figure 2 B and E
phospho PKA substrates
Representative image
tubulin
100 kDa
55 kDa
35 kDa
15 kDa
Gpr180 fl/fl
Gpr180 fl/fl
X RIP-Cre
phospho PKC substrates
Representative image
tubulin
100 kDa
55 kDa
35 kDa
15 kDa
Gpr180 fl/fl
Gpr180 fl/fl
X RIP-Cre
tu maju byt islets dva bloty pka a pkc

## Slide 8
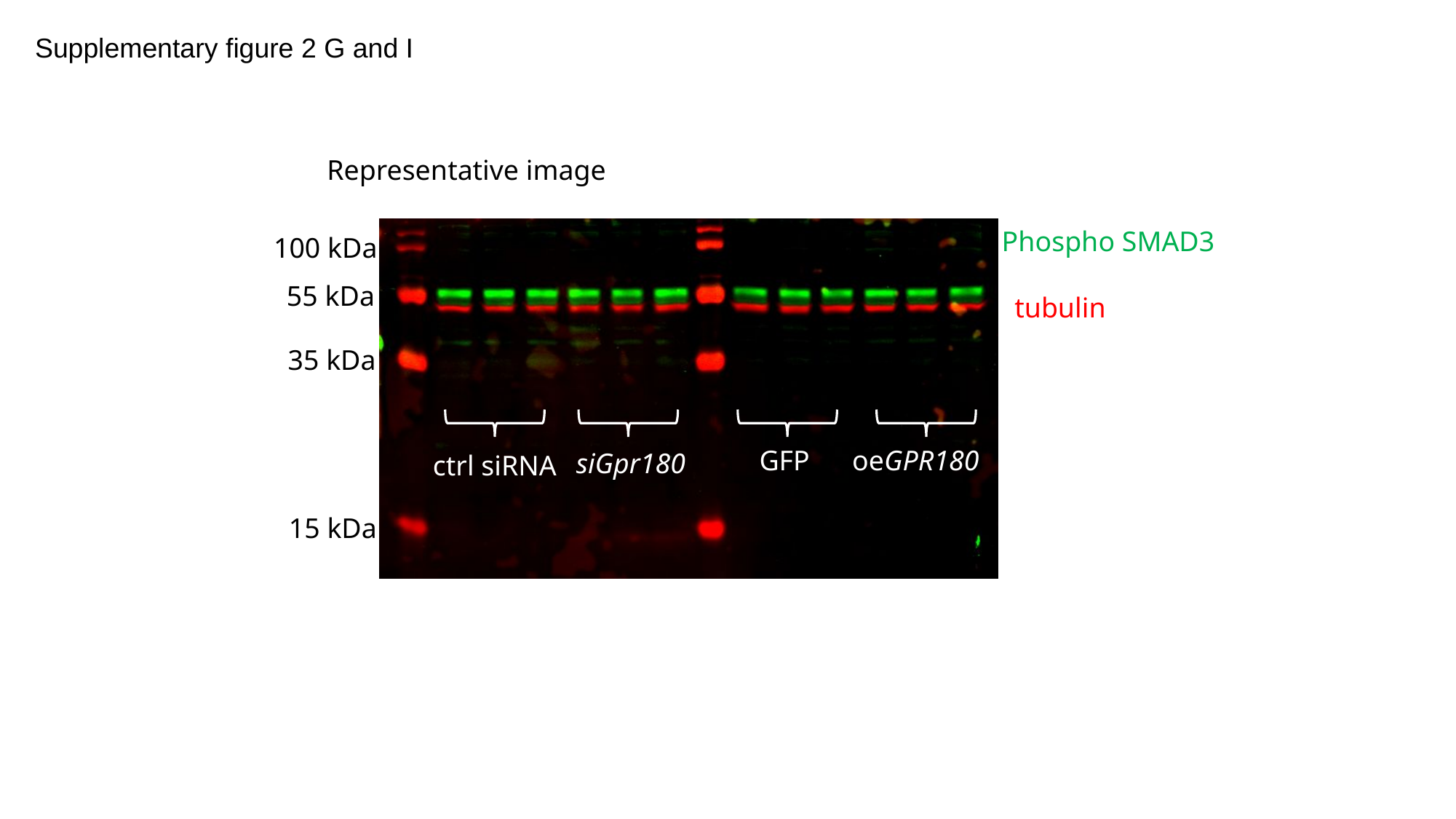

Supplementary figure 2 G and I
Phospho SMAD3
Representative image
100 kDa
55 kDa
tubulin
35 kDa
15 kDa
GFP
oeGPR180
siGpr180
ctrl siRNA

## Slide 9
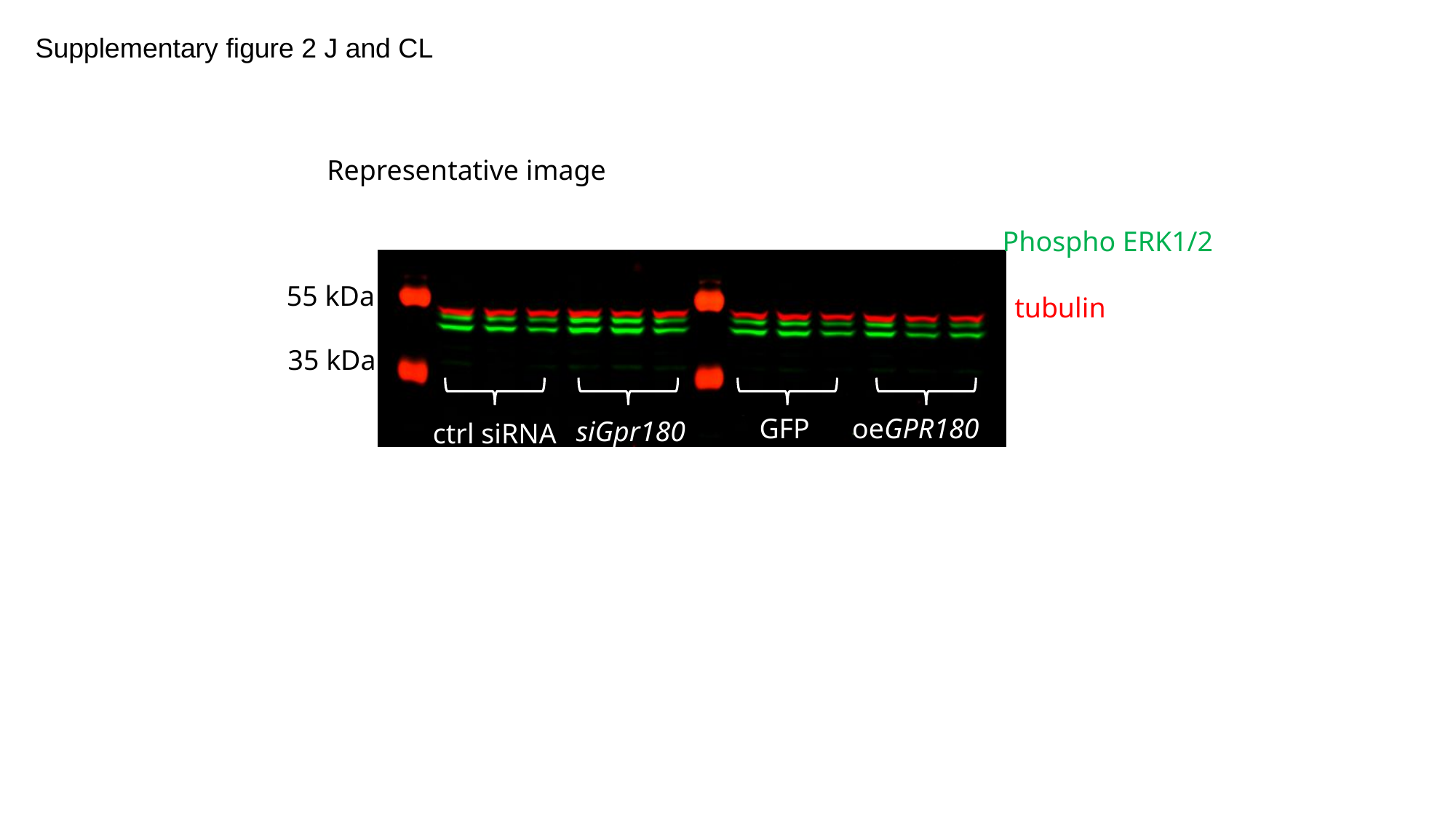

Supplementary figure 2 J and CL
Phospho ERK1/2
Representative image
55 kDa
tubulin
35 kDa
GFP
oeGPR180
siGpr180
ctrl siRNA

## Slide 10
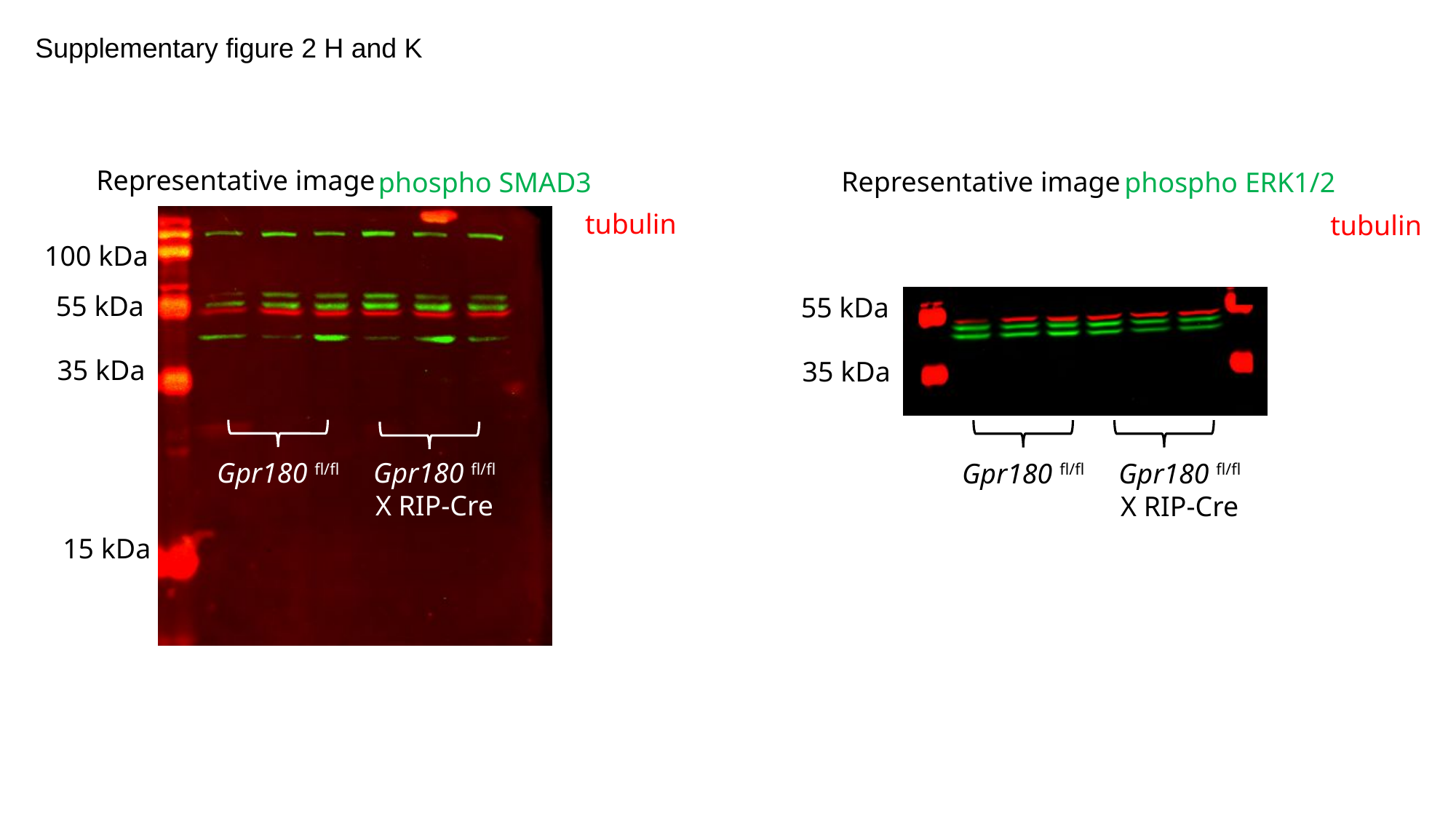

Supplementary figure 2 H and K
phospho SMAD3
Representative image
tubulin
100 kDa
55 kDa
35 kDa
15 kDa
Gpr180 fl/fl
Gpr180 fl/fl
X RIP-Cre
phospho ERK1/2
Representative image
tubulin
55 kDa
35 kDa
Gpr180 fl/fl
Gpr180 fl/fl
X RIP-Cre
